# Exon-Trapping Assay Improves Clinical Interpretation of *COL11A1* and *COL11A2* Intronic Variants in Stickler Syndrome Type 2 and Otospondylomegaepiphyseal Dysplasia

**DOI:** 10.3390/genes11121513

**Published:** 2020-12-17

**Authors:** Lucia Micale, Silvia Morlino, Annalisa Schirizzi, Emanuele Agolini, Grazia Nardella, Carmela Fusco, Stefano Castellana, Vito Guarnieri, Roberta Villa, Maria Francesca Bedeschi, Paola Grammatico, Antonio Novelli, Marco Castori

**Affiliations:** 1 Division of Medical Genetics, Fondazione IRCCS Casa Sollievo della Sofferenza, 71013 San Giovanni Rotondo, Foggia, Italy; s.morlino@operapadrepio.it (S.M.); annalisa3.schirizzi@libero.it (A.S.); g.nardella@operapadrepio.it (G.N.); c.fusco@operapadrepio.it (C.F.); v.guarnieri@operapadrepio.it (V.G.); m.castori@operapadrepio.it (M.C.); 2 Laboratory of Medical Genetics, IRCCS-Bambino Gesù Children’s Hospital, 00146 Rome, Italy; emanuele.agolini@opbg.net (E.A.); antonio.novelli@opbg.net (A.N.); 3 Unit of Bioinformatics, Fondazione IRCCS Casa Sollievo della Sofferenza, 71013 San Giovanni Rotondo, Foggia, Italy; s.castellana@operapadrepio.it; 4 Medical Genetics Unit, Fondazione IRCCS Ca’ Granda Ospedale Maggiore Policlinico, 20122 Milano, Italy; roberta.villa@policlinico.mi.it (R.V.); mariafrancesca.bedeschi@policlinico.mi.it (M.F.B.); 5 Medical Genetics Laboratory, Department of Molecular Medicine, Sapienza University, San Camillo-Forlanini Hospital, 00152 Rome, Italy; paola.grammatico@uniroma1.it

**Keywords:** *COL11A1*, *COL11A2*, stickler syndrome

## Abstract

Stickler syndrome (SS) is a hereditary connective tissue disorder affecting bones, eyes, and hearing. Type 2 SS and the SS variant otospondylomegaepiphyseal dysplasia (OSMED) are caused by deleterious variants in *COL11A1* and *COL11A2*, respectively. In both genes, available database information indicates a high rate of potentially deleterious intronic variants, but published evidence of their biological effect is usually insufficient for a definite clinical interpretation. We report four previously unpublished intronic variants in *COL11A1* (c.2241 + 5G>T, c.2809 − 2A>G, c.3168 + 5G>C) and *COL11A2* (c.4392 + 1G>A) identified in type 2 SS/OSMED individuals. The pathogenic effect of these variants was first predicted in silico and then investigated by an exon-trapping assay. We demonstrated that all variants can induce exon in-frame deletions, which lead to the synthesis of shorter collagen XI α1 or 2 chains. Lacking residues are located in the α-triple helical region, which has a crucial role in regulating collagen fibrillogenesis. In conclusion, this study suggests that these alternative *COL11A1* and *COL11A2* transcripts might result in aberrant triple helix collagen. Our approach may help to improve the diagnostic molecular pathway of *COL11*-related disorders.

## 1. Introduction

Stickler syndrome (SS) is a hereditary connective tissue disorder affecting hearing, vision, and the musculoskeletal system, and has an estimated incidence of 1:7500 to 10,000 births [1,2,3,4]. Its clinical presentation is variable and mainly includes early onset, severe myopia, vitreous changes, retinal detachment, high-frequency neurosensorial hearing loss, midfacial underdevelopment, palatal cleft with or without Pierre Robin sequence, mild spondylo-epiphyseal dysplasia primarily affecting the femoral heads, premature osteoarthritis, and joint hypermobility [5,6]. Clinical variability mirrors marked genetic heterogeneity. 

Type 1 SS (MIM #108300), representing the 80–90% of the cases, is characterized by membranous vitreous changes and is caused by heterozygous variants in *COL2A1* encoding type II collagen [7]. Type 2 SS (MIM #604841) affects the remaining 10–20% cases and is associated with monoallelic variants in *COL11A1* [8], which codes the alpha (α) 1 chain of type XI collagen. The beaded vitreous phenotype classically distinguishes SS type 2 from SS type 1 [1,8,9]. A non-ocular phenotype resembling SS has been also described in association with heterozygous or biallelic variants in *COL11A2*, encoding collagen XI α2 chains. This condition, currently named otospondylomegaepyphyseal dysplasia (OSMED), was formerly labeled as type 3 SS, or non-ocular SS [10,11]. More rarely, autosomal recessive forms of SS have been described in association with biallelic variants in *COL9A1, COL9A2, COL9A3, LOXL3,* and, more recently, *PLOD3* [12,13,14,15,16,17]. A single patient with ocular features suggestive of SS and additional findings has been described with biallelic variants in *LRP2* [18].

The *COL11A1* and *COL11A2* human genes code for the α1 and α2 chains of (pro)collagen and mature heterotrimer collagen of type XI, which is an extracellular minor fibrillar collagen. Both (pro)collagen XI α1 and α2 are present in the inner ear, hyaline cartilage, and nucleus pulposus of the intervertebral disks [19,20]. Collagen XI α1 and α2 chains play an important role in fibrillogenesis by interacting with other components of the extracellular matrix, a fact demonstrating the pleiotropic nature of type 2 SS and OSMED [19]. At the variance with the collagen XI α1 chain, the collagen XI α2 chain is not expressed in the vitreous, and this explains the absence of significant eye changes in individuals with deleterious *COL11A2* variants. Usually, nonsense, frameshift, and missense heterozygous variants in *COL11A1* and *COL11A2* are considered deleterious due to their direct effect on collagen synthesis, secretion, fibril assembly, or turnover [21]. Similarly, intronic variants affecting the mRNA splicing machinery might exert their pathogenic effect on transcription, messenger RNA (mRNA) processing, and translation [22]. This phenomenon is presumably not uncommon in *COL11A1* and *COL11A2*, as at least 49 and 12 intronic variants potentially affecting the splicing have been reported in the *COL11A1* (https://databases.lovd.nl/shared/variants/*COL11A1*, Leiden Open Variation Database) and *COL11A2* (https://databases.lovd.nl/shared/genes/*COL11A2*) variant databases, respectively. However, the presumed translational effect of these nucleotide changes is rarely investigated, with a corresponding lack of information and clinical interpretation. Here, we report four unpublished *COL11A1* and *COL11A2* splice site variants in four *COL11*-pathy families. An exon-trapping assay was used to verify the in silico predictions on splicing machinery. 

## 2. Materials and Methods 

### 2.1. Patients’ Enrollment

Subjects were enrolled after obtaining written informed consent for publishing pictures and clinical and molecular data. This study was performed in accordance with the 1984 Helsinki declaration and subsequent versions. All samples were obtained and all evaluations were performed as part of standard clinical diagnostic activities of the involved institution, including the exon-trapping assay. Hence, institutional review board (IRB) approval was not requested.

### 2.2. Sample Preparation and Next-Generation Sequencing Analysis 

Genomic DNA was extracted from the individual’s peripheral blood leucocytes by using a Bio Robot EZ1 (Qiagen, Hilden, Germany) according to the manufacturer’s instructions. DNA was quantified with a Qubit spectrophotometer (Thermo Fisher Scientific, Waltham, MA, USA). Probands’ DNA first underwent sequencing with a SureSelect gene panel (Agilent Technologies, Boulder, CO, USA) designed to selectively capture known genes associated with the various subtypes of SS, including *COL11A1* (HUGO Gene nomenclature committee, *HGNC: 2186, NM_001854*), *COL11A2* (*HGNC: 2187, NM_080680*), *COL2A1* (*HGNC: 2200, NM_001844*), *COL9A1* (*HGNC: 2217, NM_001851*), *COL9A2* (*HGNC: 2218, NM_001852*), *COL9A3* (*HGNC: 2219, NM_001853*), *LOXL3* (*HGNC: 13869, NM_032603*), *LRP2* (*HGNC: 6694, NM_004525*)*,* and *PLOD3* (*HGNC: 9083, NM_001084*)*,* according to the current nosology. Libraries were prepared using the SureSelect target enrichment kit (Agilent Technologies, Boulder, CO, USA) following the manufacturer’s instructions. Targeted fragments were then sequenced on a MiSeq/NextSeq 500 sequencer (Illumina, San Diego, CA, USA) using a MiSeq Reagent kit V3 or NextSeq 500 mid-output kit V2.5 (300-cycle flow cell). Reads were aligned to the GRCh37/hg19 reference genome by Burrows-Wheeler Aligner (BWA) (v.0.7.17). BAM files were sorted by SAMtools (v.1.7) and purged from duplicates using Mark Duplicates from the Picard suite (v.2.9.0). Mapped reads were locally realigned using GATK 3.8. Reads with mapping quality scores lower than 20 or with more than one-half nucleotides with quality scores less than 30 were filtered out. The GATK’s Haplotype Caller tool was used to identify variants. Variants were functionally annotated by implementing the ANNOVAR program on the NCBI RefSeq transcript reference system, with information about allelic frequency (1000 Genomes, dbSNP v151, GO-ESP 6500, ExAC, TOPMED, gnomAD, NCI60, COSMIC), reported or computationally estimated pathogenicity (Varsome, ClinVar, HGMD, LOVD, or SIFT, Polyphen2, LRT, MutationTaster, MutationAssessor, FATHMM, PROVEAN, VEST3, MetaSVM, MetaLR, M-CAP, CADD, DANN, fathmm-MKL, Eigen, GenoCanyon), and genomic site conservation (fitCons, GERP++, phyloP100way, phyloP20way, phastCons100way vertebrate, phastCons20way mammalian, SiPhy 29way). Selected variants were interpreted according to the American College of Medical Genetics and Genomics/Association for Molecular Pathology (ACMGG/AMP) [23].

Variants sorted as “benign” and “likely benign” were excluded. Remaining variants were classified, based on the American College of Medical Genetics (ACMG) standards and guidelines for the interpretation of sequence variants, as pathogenic, likely pathogenic, or of uncertain significance by using the following prioritization criteria: (i) null variant (nonsense, frameshift, deletion, insertion, canonical ±1 or ±2 splice site) in genes previously described as disease-causing by haploinsufficiency or loss of function; (ii) variant located in a mutational hot spot and/or critical and well-established functional domain; (iii) variant absent in allele frequency population databases; (iv) variant reported in allele frequency population databases, but with a minor allele frequency (MAF) significantly lower than expected for the disease; (v) variant annotated as pathogenic in ClinVar and/or LOVD; (vi) variant co-segregation with disease in multiple affected family members; (vii) well-established in vitro or in vivo functional studies supportive of a damaging effect on the gene or gene product. Common (MAF > 0.01) and synonymous variants were discarded.

### 2.3. Sanger Sequencing

*COL11A1* (HUGO Gene Nomenclature Committee, HGNC ID: 2186) and *COL11A2* (HGNC ID: 2187) variants identified by next-generation sequencing (NGS) were confirmed by Sanger sequencing and resequenced in independent experiments. Primer sequences are reported in supplementary Table S1. The amplified products were subsequently purified by using the ExoSAP-IT PCR Product Cleanup Reagent (Thermo Fisher Scientific, Waltham, MA, USA) and sequenced by using the BigDye Terminator v1.1 sequencing kit (Thermo Fisher Scientific, Waltham, MA, USA), purified using DyeEx plates (Qiagen, Hilden, Germany), and resolved on an ABI Prism 3130 Genetic Analyzer (Thermo Fisher Scientific, Waltham, MA, USA). Sequences were analyzed using the Sequencer software (Gene Codes, Ann Arbor, MI, USA). In individuals with positive family history, segregation from the affected parent was carried out by Sanger sequencing. In apparently *de novo*, naturally conceived cases, paternity was tested by microsatellite analysis.

### 2.4. In Silico Variant Analysis

Splice-site variants were classified on the basis of in silico splice predictors, including NetGene2 (http://www.cbs.dtu.dk/services/NetGene2/), Berkeley Drosophila Genome Project (BDGP, http://www.fruitfly.org/seq_tools/splice.html), and Human Splicing Finder (HSF, http://www.umd.be/HSF3/).

### 2.5. Variant Designation

Nucleotide variant nomenclature follows the format indicated in the Human Genome Variation Society (HGVS, http://varnomen.hgvs.org/) recommendations. The DNA variant numbering system refers to cDNA. Nucleotide numbering uses +1 as the A of the ATG translation initiation codon in the reference sequence, with the initiation codon as codon 1.

### 2.6. Minigene Assay

The in vitro splicing assay was carried out using a pSPL3 exon-trapping vector provided by Tompson and Young [24]. Briefly, the pSPL3 vector contains a small artificial gene composed of an SV40 promoter, an exon A–intron–exon B sequence with functional splice donor and acceptor sites, and a late polyadenylation signal. For *COL11A1* c.2241 + 5G>T, wild-type and mutant *COL11A1* exon 26 was directly PCR amplified from Individual #1 with specific primers containing additional XhoI (forward) and BamHI (reverse) restriction sites. The PCR reaction amplified the entire exon 26 sequence plus an additional 312 bp (5′) and 466 bp (3′) of the flanking intronic regions. For *COL11A1* c.2809 − 2A>G, a DNA fragment of 750 bp, including the *COL11A1* exon 37, 336 bp (5′), and 360 bp (3′) of the flanking intronic regions, was amplified from Individual #2 with specific primers. For *COL11A1* c.3168 + 5G>C, an 820 bp genomic DNA fragment that comprises *COL11A1* exon 41 and its flanking sequences (including exon 40, intron 40, and parts of introns 39 and 41) was amplified by PCR with primers containing the appropriate restriction enzyme sites from Individual #3′s DNA. For *COL11A2* c.4392 + 1G>A, the wild-type and mutant *COL11A2* region including both the exon 60 and corresponding flanking sequences (including exon 59, intron 59, and parts of introns 58 and 60) was directly PCR amplified from Individual #4 with specific primers. In the last two vectors, we cloned two exons because the intron (introns 40 or 59, respectively) included among these is short. All primer sequences are listed in Table S1.

After PCR amplification, PCR products were purified and subjected to restriction enzyme digestion, and were then inserted into pSPL3 exon-trapping vector. All minigene constructs were then Sanger sequenced to verify the correctness of the wild-type and mutant DNA fragments. Vectors containing either wild-type or variant sequences or no insert (empty vector) were transfected into HEK 293T cells in triplicate using Lipofectamine^®^ LTX (Thermo Fisher Scientific, USA), according to the manufacturer’s instructions. Total RNA was harvested 48 h post-transfection using an RNeasy Mini Kit (Qiagen, Hilden, Germany), treated with RNase-DNase free (Qiagen, Hilden, Germany), quantified by Nanodrop (Thermo Fisher Scientific, Waltham, MA, USA), and reverse-transcribed using a QuantiTect Reverse Transcription Kit (Qiagen, Hilden, Germany), according to the manufacturer’s instructions. The cDNA was PCR amplified using vector-specific primers (V1-forward and V1-reverse). The primers used for cDNA amplification are given in Table S1. The amplified fragments were visualized on a 1% agarose gel and subsequently Sanger sequenced.

## 3. Results

### 3.1. Individual #1: Clinical Report

Individual #1 was a 27-year-old Italian woman referred to Medical Genetics consultation for myopia and musculoskeletal pain, as well as an original suspicion of Ehlers–Danlos syndrome, by a secondary center physician. She was the second child of a father with moderate myopia, who died at 55 years due to cerebral neoplasia, and a mother affected by hydroadenitis suppurativa and lichen planus pilaris. This individual had an older sister with mild myopia. Since the age of six years, she presented myopia, which was of −13.0 diopters on the left eye and −12.0 diopters on the right eye at the time of examination. There was not any evidence of vitreous degeneration. She also reported recurrent knee dislocations and chronic pain in the shoulders since adolescence. Due to chronic back pain, lumbar spondylolisthesis was noted. At 24 years of age, audiometry revealed moderate, bilateral neurosensorial hearing loss predominantly involving high frequencies and, at 26 years, a heart ultrasound disclosed mild mitral valve prolapse. Physical examination showed a flattened malar region, hypoplastic nasal bridge, and bilateral epicanthus. The anthropometry and pattern of joint mobility were within normal range. Brachydactyly type D was also evident on the left hand. Individual #1 was the mother of a four-year-old boy with myopia (−3.0 diopters on the left eye and −4.0 diopters on the right eye), mild bilateral neurosensorial hearing loss, mild hypoplasia of the malar region, hypertrophic upper labial frenulum, and mild diastasis of the upper incisors. Palate, stature, and psychomotor development were normal.

#### Individual #1: Molecular Findings

**Figure 1:**
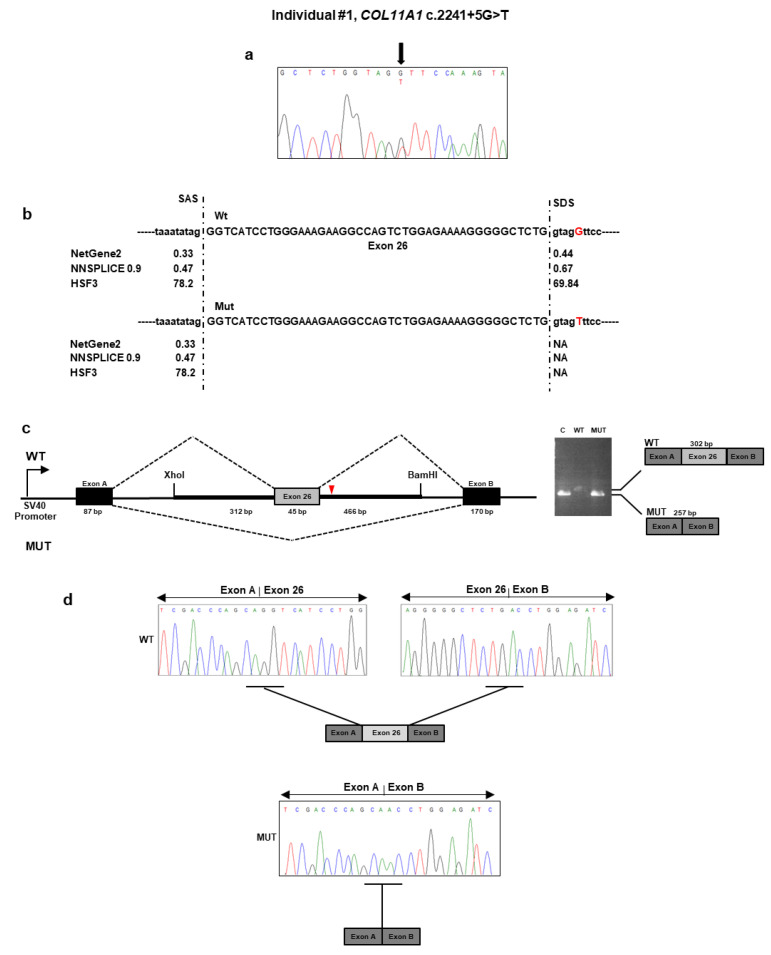
Molecular findings of individual #1. (**a**) Electropherogram showing DNA sequencing analysis of the PCR product amplified with primers targeting exon 26 and flanking intronic regions of *COL11A1.* Nucleotide sequences are provided. (**b**) Splicing prediction of the scores of the intronic variant indicate the potential alteration of the splicing process. SAS: splice acceptor site; SDS: splice donor site; NA: not available; WT: wild type; MUT: mutated. (**c**) Analysis of the c.2241 + 5G>T variant using the minigene construct. The position of the variant site and fragment containing exon 26 and its adjacent introns are indicated. Analysis of mRNA from transfected HEK293 cells via reverse transcriptase (RT)-PCR (on the gel, C: empty vector; WT: wild type; MUT: mutated) and (**d**) direct sequencing.

NGS analysis performed on DNA from individual #1 identified the heterozygous c.2241 + 5G>T variant located in the intron 26 of *COL11A1* gene (Table 1). No other potential candidate variants were detected in the remaining genes. The c.2241 + 5G>T variant was absent from all applied population and disease-specific databases, suggesting that it is novel. The result was confirmed by direct Sanger sequencing of individual #1 DNA (Figure 1a). The variant was also detected in her affected son’s DNA. To evaluate the pathogenicity of this variant, we used several in silico splice-site prediction programs, such as NetGEne2, NNSPLICE, and Human Splice Finder3. These analyses predicted that the nucleotidic change weakened the donor spliced site (Figure 1b). To validate these predictions, we employed a minigene-based splice assay. The minigenes that carried the wild-type sequence and c.2241 + 5G>T variant were transfected into HEK293 cell line. The reverse transcriptase (RT)-PCR analysis performed on total RNA extracted from the transfected cells detected a 302 bp band in cells that were transfected with the wild-type region and a 257 bp band in both cells transfected with the empty vector and the mutant plasmid (Figure 1c). Direct sequencing of the RT-PCR products revealed that the larger fragment contained the exon 26, as expected. In contrast, the shorter fragment lacked the exon 26 (Figure 1d). The exon 26 skipping may lead to the synthesis of a shorter collagen XI α1 chain defective of the 15 residues (733–747) of the α1 helical region. The variant has been submitted to LOVD (https://databases.lovd.nl/shared/variants/0000687717#00023834, individual ID # 00311014).

### 3.2. Individual #2: Clinical Report

Individual #2 was the naturally conceived first daughter of non-consanguineous parents with negative family history. Prenatally, second trimester high-resolution ultrasound scan revealed polyhydramnios, biparietal diameter and abdominal circumference >95th percentile, length of long bones at the lower end (5th–10th centile), orbital hypertelorism, and suspected craniosynostosis. She was born at term by elective Caesarean delivery because of fetal–maternal disproportion. Her weight was 3925 g (>97th percentile), length 48.3 cm (25–50th percentile), and head circumference 37.5 cm (>97th percentile) with a wide anterior fontanelle. A flattened and underdeveloped midface, apparent exophthalmos, small nose with depressed nasal bridge, median submucous cleft palate, and micrognathia were also noticed. At birth, the echocardiogram and cerebral and abdominal ultrasound were unremarkable, except for patent foramen ovale. A brain magnetic resonance imaging (MRI) showed small focal bilateral cerebellar deep white matter heterotopia, enlarged and dysmorphic ventricular system, and apparently absent left olfactory bulb.

In the following months, ophthalmologic evaluation confirmed exophthalmos and also noted right eye strabismus, horizontal nystagmus, and myopic chorioretinopathy. Cycloplegic refraction disclosed severe myopia with −14.00 sf and −4.00 cyl 40° degree in the right eye, and −14.50 sf −3.50 cyl 70° degree in the left eye. Visually evoked potentials and an electroretinogram performed at six months of age showed residual visual capacity and retinal function. A neonatal otoacoustic emission screening test failed on the right. At four months of age, acoustic-evoked potentials demonstrated mild conductive hearing loss. She stood alone at nine months, walked unsupported at 18 months, and said her first words at 12 months, but her language was poor at 22 months (last examination). The patient was introduced early into a child neurology program due to mild developmental delay.

#### Individual #2: Molecular Findings

**Figure 2:**
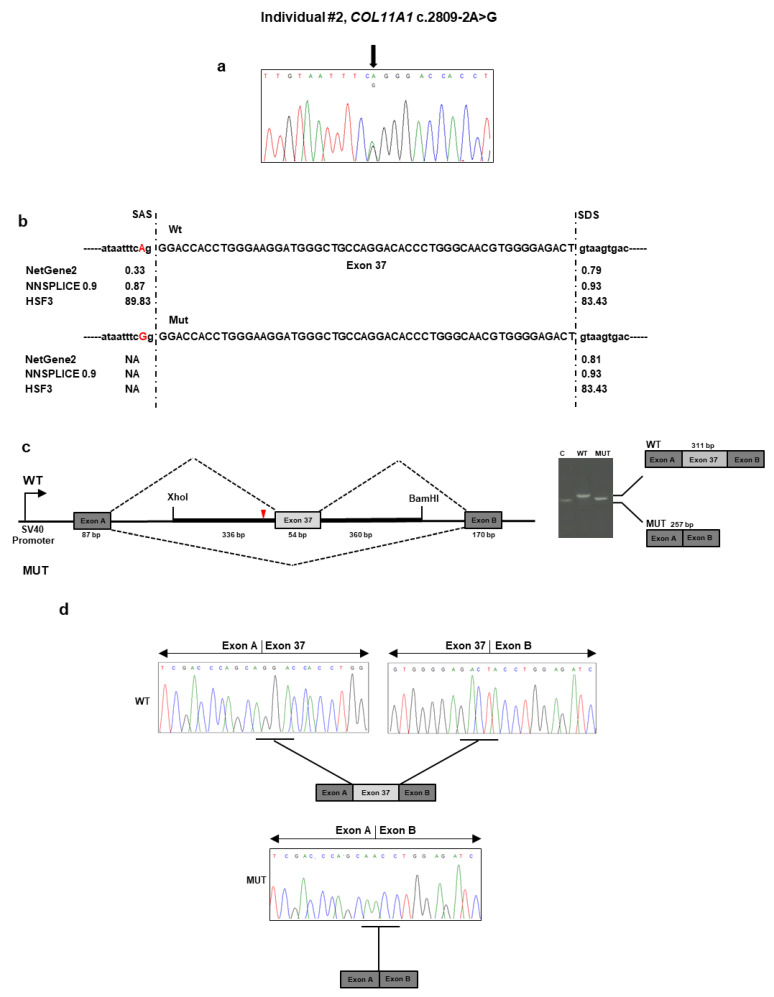
Molecular findings of individual #2. (**a**) Electropherogram of *COL11A1* exon 37 and flanking intron sequence showing the heterozygous c.2809 − 2A>G variant. Nucleotide sequences are provided. (**b**) Prediction of the scores of splice acceptor sites of *COL11A1* exon 37 of the wild-type and mutated genomic bases. (**c**) Analysis of the c.2809 − 2A>G variant using the minigene construct. The positions of the variant site and fragment containing exon 37 and its adjacent introns are indicated. Analysis of mRNA from transfected HEK293 cells via RT-PCR (on the gel, C: empty vector; WT: wild type; MUT: mutated) and (**d**) direct sequencing.

The targeted NGS platform revealed that individual #2 carried out an unpublished splice site c.2809 − 2A>G variant located in intron 36 of *COL11A1* (Table 1 and Figure 2a). This variant was previously described as “likely pathogenic” in ClinVar in a patient with SS (https://www.ncbi.nlm.nih.gov/clinvar/variation/845674/). No other candidate variants were found in the remaining genes. The result was confirmed by direct Sanger sequencing of the patient’s DNA. The variant was not detected in the patient’s parents and, therefore, was assumed to be *de novo* (Figure 2a). According to algorithms developed to predict the effect of sequence changes on RNA splicing, the variant is expected to disrupt the intron 36 splice acceptor site (Figure 2b). That loss would likely mediate exon skipping or the use of an alternative cryptic splice site nearby. To characterize the impact of the c.2809 − 2A>G variant on RNA splicing, we cloned the wild-type and mutant sequence of *COL11A1* exon 37 and flanking introns in the pSPL3 vector and transfected them into the HEK293 cell line. Analysis of the splicing products from the minigene assay revealed that cells transfected with the wild-type vector yielded the expected 311 bp band containing the exon 37 (Figure 2c). In contrast, cells transfected with the mutant vector yielded a band at 257 bp lacking the exon 37. Sequencing of all bands confirmed break points and splicing events (Figure 2d). The deletion of exon 37 was predicted to generate a shorter collagen XI α1 chain that lacks 18 residues (937–954) located in the α1 helical region. The variant has been submitted to LOVD (https://databases.lovd.nl/shared/variants/0000687726#00023834, individual ID #00311022).

### 3.3. Individual #3: Clinical Report

**Figure 3:**
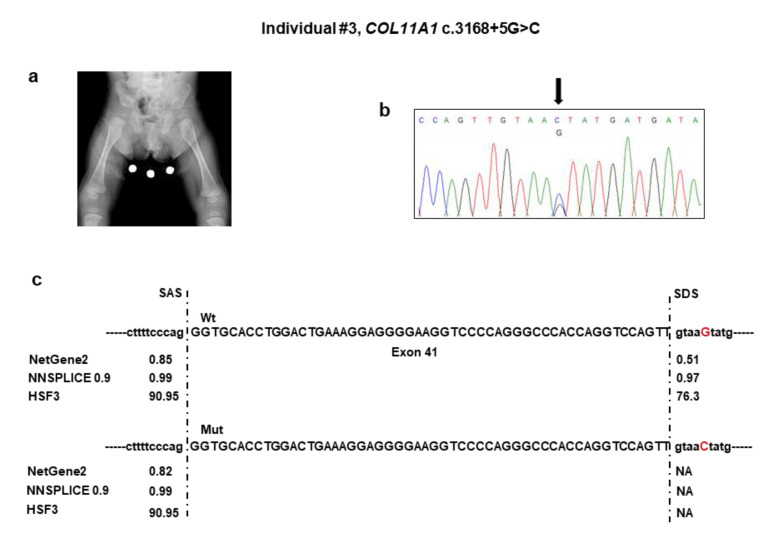

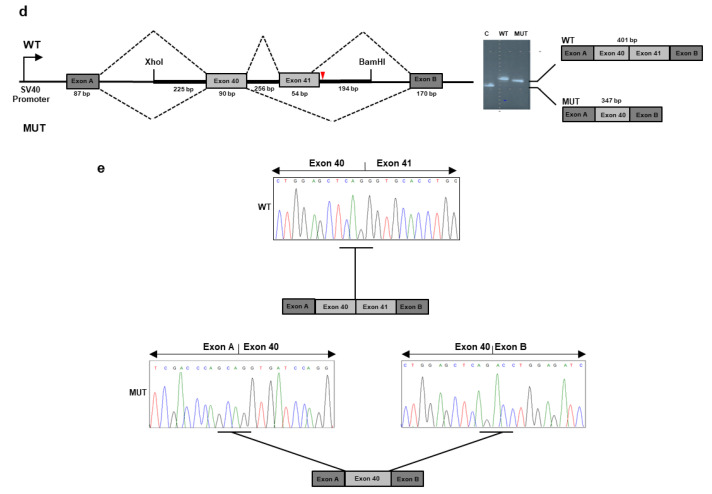
Molecular findings of individual #3. (**a**) Pelvis and femora radiograph at birth showing restricted ischiatic notch, early ossification of the proximal femoral epiphyses, enlarged distal femoral and proximal tibial epiphyses, and flared metaphyses. (**b**) Electropherogram of *COL11A1* exons 40–41 and flanking intron sequence showing the heterozygous c.3168 + 5G>C variant. Nucleotide sequences are provided. (**c**) Predictions of the scores of splice acceptor sites of *COL11A1* exon 37 of the wild-type and mutated genomic bases. (**d**) Analysis of the c.3168 + 5G>C variant using the minigene construct. The positions of the variant site and fragment containing exon 37 and its adjacent introns are indicated. Analysis of mRNA from transfected HEK293 cells via RT-PCR (on the gel, C: empty vector; WT: wild type; MUT: mutated) and (**e**) direct sequencing.

Individual #3 was a naturally conceived newborn from an uneventful pregnancy and at-term vaginal delivery with an Apgar score of 8^1^/9^5^, length of 46 cm (2nd percentile), head circumference of 33 cm (15th percentile), and weight of 2620 g (9th percentile). Soon after birth, she developed respiratory distress due to upper airway obstruction, which required admission to the neonatal intensive care unit. Here, the Pierre Robin sequence was diagnosed for the combination of micrognathia, glossoptosis, and cleft soft palate. This requested early mandibular distraction. Aspecific brain lesions attributed to the respiratory distress were detected with transfontanellar ultrasound at birth and subsequently confirmed by brain MRI, which also showed hypoplasia of the corpus callosum and buphthalmus. In addition, neonatal physical examination revealed a broad forehead with frontal bossing, ocular proptosis with bilateral buphthalmos and megalocornea (diameter 13 mm), blue sclerae, midface hypoplasia, and hypoplastic nose with anteverted nostrils. Total body radiographs at birth showed reduced ischiatic notch, precocious ossification of the proximal femoral epiphyses, broad metaphyses, especially of the lower limbs (Figure 3a), short phalanges, frontal bossing with an enlarged anterior fontanel, micrognathia, and mild platyspondyly. Auditory brainstem response test was negative, but audiological examination disclosed recurrent otitis media and conductive hearing loss. At the last examination at one year of age, psychomotor development was normal.

#### Individual #3: Molecular Findings

NGS analysis detected the c.3168 + 5G>C variant in the intron 41 of *COL11A1* (Table 1). No other candidate variants were found in the remaining genes. Sanger sequencing confirmed the variant in the patient (Figure 3b) and documented that it was absent in both parents. The c.3168 + 5G>C variant was not reported in major databases and, therefore, is novel, and is expected to disrupt the intron 41 splice donor site (Figure 3c). The effect of the splice variant at the mRNA level was evaluated by using the minigene assay. PCR amplification and Sanger sequencing of wild-type and mutant cDNA revealed two products of different sizes, which is the result of exon 41 skipping in the mutated sequence (347 bp) compared to the wild-type control (401 bp) due to disruption of the intron 41 splice donor site (Figure 3d,e). The exon 41 skipping lead to a Collagen XI α1 chain shorter by 18 residues (1039–1056) in the α1 helical region. The variant has been submitted to LOVD (https://databases.lovd.nl/shared/variants/0000687720#00023834, individual ID #00311016).

### 3.4. Individual #4: Clinical Report

**Figure 4:**
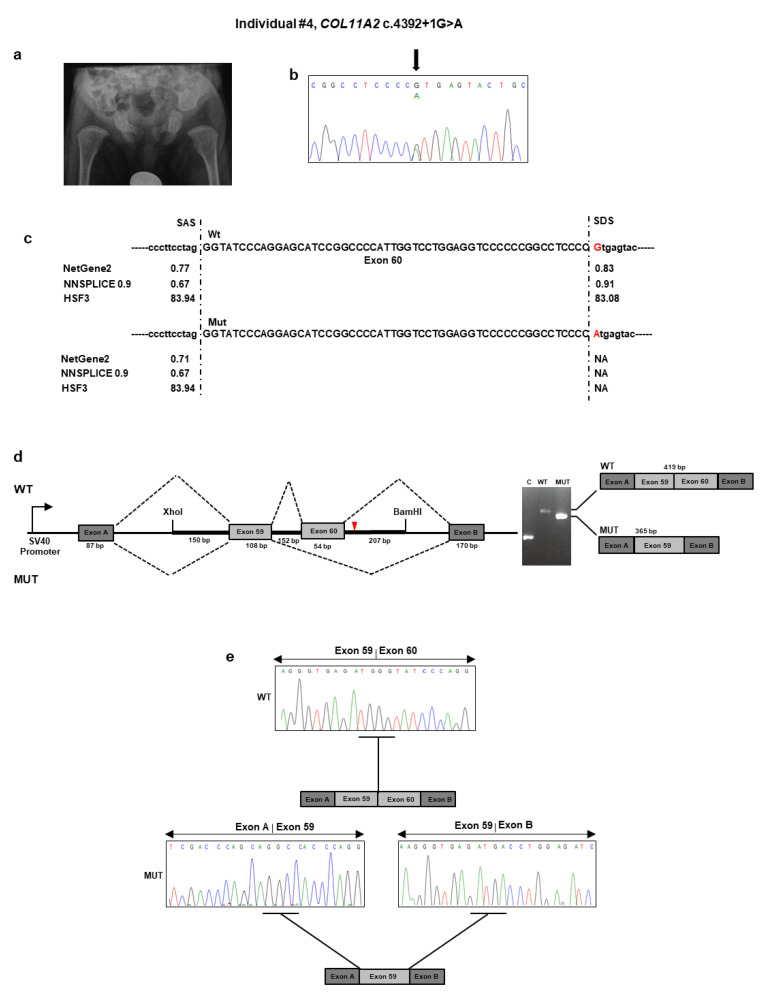
Molecular findings of individual #4. (**a**) Pelvis radiograph at birth. Note the broad femoral metaphyses and enlarged sciatic notch. (**b**) Electropherogram of *COL11A2* exon 37 and flanking intron sequence showing the heterozygous c.4392 + 1G>A variant. Nucleotide sequences are provided. (**c**) Prediction of the scores of splice acceptor sites of *COL11A2* exon 60 of the wild-type and mutated genomic bases. (**d**) Analysis of the c.4392 + 1G>A variant using the minigene construct. The positions of variant site and fragment containing exon 60 and its adjacent introns are indicated. Analysis of mRNA from transfected HEK293 cells via RT-PCR (on the gel, C: empty vector; WT: wild type; MUT: mutated) and (**e**) direct sequencing.

This subject was a nine-month-old boy, born from heterologous in vitro fertilization to a 33-year-old woman and an anonymous father. Third-trimester prenatal ultrasound revealed polyhydramnios. Delivery was at term with a neonatal weight of 2500 g (3rd percentile), length of 45 cm (<1st percentile), and head circumference of 34 cm (25° percentile). The Apgar score was 7^1^/9^5^. At birth, the neonatologist noted hypotonia, micro/retrognathia, and U-shaped palatal cleft. Due to ineffective deglutition, percutaneous endoscopic nutrition by gastrostomy was requested shortly after birth and lasted for seven months. At that time, profound bilateral neurosensorial hearing loss was diagnosed and corrected with external prostheses. The patient sat alone at 7.5 months. At nine months of age, length was 65 cm (<1st percentile), weight was 5900 g (<1st percentile), and head circumference was 43 cm (1st percentile). Physical examination revealed frontal bossing with incompletely closed anterior fontanel, flattening of the malar region, hypoplastic nasal tip and bridge, anteverted nares, and retrognathia. A small congenital melanocytic nevus was evident at the abdomen. The rest of the examination was normal. Ophthalmological examination gave normal results. A pelvis radiograph at birth demonstrated broad femoral proximal metaphyses and an enlarged sciatic notch (Figure 4a). His mother presented with comparable facial features (i.e., flattened malar region, hypoplastic nasal bridge, anteverted nares). When she was a child, the mother underwent maxillofacial surgery for palatal cleft. She did not refer to any ocular or audiological problems. Stature was normal.

#### Individual #4: Molecular Findings

NGS analysis found the unpublished c.4392 + 1G>A variant in intron 60 of *COL11A2* (Table 1 and Figure 4b). This variant was previously described as “pathogenic” in ClinVar in a patient with OSMED (https://www.ncbi.nlm.nih.gov/clinvar/variation/17120/). The variant was detected in the affected mother, as confirmed by a co-segregation study. Bioinformatics tools suggested that the mutant sequence might break the original splice site and affect pre-mRNA splicing (Figure 4c). To confirm the predicted splicing defect of the mutated *COL11A2* mRNA, we then analyzed *COL11A2* transcripts expressed from mini-constructs containing the mutated or wild-type alleles in HEK293 cells. After RT-PCR and gel separation of cDNA amplicons, we observed a size difference when comparing products derived from the wild-type and mutated constructs (Figure 4d). The size difference was consistent with a predicted altered splicing due to the c.4392 + 1G>A variant. Purification of the gel-separated cDNA amplicons followed by sequence analysis confirmed that transcripts from the mutated *COL11A2* allele lacked 54 bp of exon 60, leading to the synthesis of a collagen XI α2 chain shorter by 18 aa (1447–1464), located in the α2 helical region (Figure 4e). The variant has been submitted to LOVD (https://databases.lovd.nl/shared/variants/0000687722#00024025, individual ID # 00311018).

## 4. Discussion

Here, we reported the clinical description, genomic investigation, and functional data of four families with type 2 SS/OSMED due to deleterious variants affecting intronic splicing sites of *COL11A1* and *COL11A2*. Two variants (i.e., c.2241 + 5G>T and c.3168 + 5G>C in *COL11A1*) were novel, while the other two (i.e., c.2809 − 2A>G in *COL11A1* and c.4392 + 1G>A in *COL11A2*) were unpublished and reported in ClinVar without any data on their actual outcome at the mRNA level. In all but one case (Individual #1), the overall clinical picture was suggestive of a *COL2/COL11*-pathy shortly after birth at prompt clinical genetics consultation. In the remaining patient, who is the unique adult in this case series, the suspicion of SS was considered more appropriate than Ehlers–Danlos syndrome after tertiary center consultation and years of musculoskeletal pain. In all circumstances, molecular testing was carried out with a virtual multigene panel designed for a wide range of partially overlapping hereditary connective tissue disorders. The potential effect of the identified intronic variants in generating abnormal alternative isoforms was predicted and then demonstrated with an exon-trapping assay in all cases.

According to the ACMG guidelines, available data demonstrating the effects of intronic variants on splicing are recommended for variants affecting both the canonical +/−1 or 2 splice site (intronic variants within GT and AG splice site pairs) and non-canonical splice sites (rarer GT and AG intron splice site pairs) [25,26]. Concerning canonical splice-site variants, current application of the PVS1 criterion may be eased by in vitro assays supporting in silico predictions, especially for private variants or variants with scanty database and literature data [26]. The PVS1 criterion refers to the null variant (nonsense, frameshift, canonical ±1 or 2 splice sites, initiation codon, single or multiexon deletion) in a gene where loss of function is a known mechanism of disease. For intronic variants affecting potential non-canonical splice sites or generating cryptic splice donor or acceptor sites, the results of in vitro assays might help in the correct application of the PS3/BS3 (PS3: well-established in vitro or in vivo functional studies supportive of a damaging effect on the gene or gene product; BS3: in-frame deletions/insertions in a repetitive region without a known function) versus PVS1 criteria [26]. For these variants, the lack of any experimental proof on the predicted post-genomic effects generally limits their clinical interpretation to “variants of unknown significance”. Although RNA-splicing assays cannot predict the effect of the intronic variant at the protein level, they can be easily standardized and validated with unaffected controls for a potential clinical use.

In clinics, the choice of the most effective way to obtain in vitro data on intronic variants is critical. In fact, not all genes are expressed in peripheral blood and not all conditions allow sampling of other tissues according to the disease-specific clinical pathway. In addition, other accessible tissues might not be effective for obtaining reliable data for functional assays. For example, fresh tissue sampling for obtaining a patient’s mRNA might be suboptimal, as transcripts are not stable within tissues, and abnormal transcripts can be quickly degraded by nonsense-mediated mRNA decay. Recently, exon-trapping assay approaches have been established as a relatively fast and accurate tool to characterize potential splicing aberrations and to predict their effects at the transcriptional level [21,27,28,29]. These constructs contain a genomic fragment from the gene of interest that includes the exon(s) and partial or entire flanking intronic regions, and they are able to express wild-type or aberrant pre-mRNAs by transient transfection, thus providing a rapid assay for evaluating the transcriptional effect of intronic variants.

Here, we molecularly explored the transcriptional impact of four unpublished intronic variants in *COL11A1* (c.2241 + 5G>T, c.2809 − 2A>G, c.3168 + 5G>C) and *COL11A2* (c.4392 + 1G>A) associated with type 2 SS/OSMED. The *COL11A1* c.2241 + 5G > T and c.3168 + 5G>C variants were predicted to abolish the donor splice site of exons 26 and 41, respectively. On the contrary, the c.2809 − 2A>G *COL11A1* variant was predicted to disrupt the acceptor splice site of exon 37. We confirmed aberrant splicing of exons 26, 37, and 41 due to c.2241 + 5G>T, c.2809 − 2A>G, and c.3168 + 5G>C *COL11A1* variants, respectively, by using an exon-trapping assay. Aberrant splicing leading to skipping of exons 26, 37, and 41 would result in an in-frame deletion and collagen XI α1 chains lacking residues 733−747 (15 aa), 937−954 (18 aa), 1039–1056 (18 aa), respectively. Kohmoto et al. (2015) reported an SS patient carrying a heterozygous intronic *COL11A1* variant affecting the residue c.3168 + 5G with a different substitution (G>A), which results in the skipping of the entire exon 41, as demonstrated by an exon trapping strategy [30]. Similar results from a minigene assay have been previously reported in SS for a small insertion recurring in intron 41 of *COL11A1* [31,32]. Based on all of this experimental evidence, we speculate that intron 41 may be a mutational hot spot for splicing variants in SS Type 2. The c.4392 + 1G>A *COL11A2* splice site variant was predicted to result in the synthesis of a shorter collagen XI α2 lacking 18 aa (1447–1464). Our exon-trapping assay confirmed such a prediction at the mRNA level and, hence, the efficacy of such an in vitro tool in confirming in silico prediction of intronic splicing variants in *COL11A2*.

The lacking *COL11A1* and *COL11A2* residues are located in the α-triple helical region of collagen fiber. Each collagen is usually made of three different polypeptides, designated as α1, α2, and α3, and encoded by *COL11A1, COL11A2*, and *COL2A1*, respectively. The three α chains form the triple helical part of the molecule and contribute differentially in the proper formation and function of bones, cartilage, and the ocular and auditory systems [33,34]. Similarly to the other fibrillar collagens, it is composed of repeating peptide triplets of “glycine-X-Y”, where X and Y can be any amino acid, most often proline and hydroxyproline, respectively. The lack of the glycine-X-Y triplets encoded by exons 26, 37, and 42 of *COL11A1* and exon 60 of *COL11A2* could result in altered collagen molecules that generate an aberrant triple helix collagen with a presumed dominant negative effect [32].

Analysis of whole RNA from individuals harboring the *COL11A1* and *COL11A2* variants would better define the splicing defects resulting from these variants. Unfortunately, *COL11A1* and *COL11A2* are not expressed in peripheral blood, and other tissues were not available for these individuals. Reasonably, it is possible that other splicing events occur in addition to exon skipping but could not be detected with the current minigene design. Since only parts of introns flanking the exons carrying the identified *COL11A1* and *COL11A2* variants were tested with our approach, we were unable to determine whether these variants might also activate a cryptic donor/acceptor site(s) somewhere outside the investigated sequence to generate larger aberrant exons.

In conclusion, we demonstrated that the exon-trapping essay can be effectively applied to test the predicted splicing effect of the *COL11A1* and *COL11A2* intronic variant. Given the high rate of potentially deleterious variants falling in intronic sequences of such genes, our results prompt the integration of such investigations as second-tier analyses in the diagnostic workflow of laboratories with interest in collagen-related hereditary disorders.

## Figures and Tables

**Table 1:** Characteristics of *COL11A1* and *COL11A2* variants identified in this study. Abbreviations are as follows: chr: chromosome; NA: not available; VUS: variant uncertain significance; PM2: variant not found in gnomAD exomes or genomes; PVS1: null variant; PP5: ClinVar classifies this variant as “Likely Pathogenic” or “Pathogenic”, rated one star, criteria provided, single submitter, with one submission; M: moderate; VS: very strong; S: supporting.

Individual	1	2	3	4
**Gene**	*COL11A1 (HGNC: 2186)*	*COL11A1 (HGNC: 2186)*	*COL11A1 (HGNC: 2186)*	*COL11A2 (HGNC: 2187)*
**Variant**	c.2241+5G>T chr1: g.103462631G>T	c.2809-2A>G chr1: g.103435830A>G	c.3168+5G>C chr1: g.103427417G>C	c.4392+1G>A chr6: g.33134289G>A
**Intron number**	26	36	41	60
**rs-ID (dbSNP)**	NA	NA	NA	rs750995470
**Parental origin**	NA	De novo	De novo	Mother
**LOVD-ID**	00311014	00311022	00311016	00311018
**Frequency**				
**gnomAD-Total**				
**Allele count**	NA	NA	NA	3
**Allele number**	NA	NA	NA	249.798
**No. of homozygotes**	NA	NA	NA	0
**Allele freq.**	NA	NA	NA	0.000012
**Varsome**				
**Verdict**	VUS	Pathogenic	VUS	Pathogenic
**Rules**	PM2	PVS1 PM2 PP5	PM2	PVS1 PM2 PP5
**Strength**	M	VS M S	M	VS M M

## Supplementary Materials

The following are available online at https://www.mdpi.com/2073-4425/11/12/1513/s1, Table S1: Sequences of *COL11A1* and *COL11A2* primers used in this study.
