# Supplementary material for: Exon-Trapping Assay Improves Clinical Interpretation of COL11A1 and COL11A2 Intronic Variants in Stickler Syndrome Type 2 and Otospondylomegaepiphyseal Dysplasia
Source: Genes (Basel). 2020 Dec 17;11(12):1513. doi: 10.3390/genes11121513 (PMC7766184; doi:10.3390/genes11121513)
Supplement: Supplementary file 1 [file genes-11-01513-s001.pdf]

**Table S1. Sequences of *COL11A1* and *COL11A2* primers used in this study.**			
**Name**	**Sequence**	**Size**	**Use**
pSPL3COL11A1-c.2241+5G>T-F	ctgactgaCTCGAGctttaattgaggctggggttgaa	823	Cloning
pSPL3COL11A1-c.2241+5G>T-R	tcagtcagGGATCCaaaatgatcctacccctgctact		
pSPL3COL11A1-c.2461-2A>G-F	ctgactgaCTCGAGtcatttgtgaagtgctaaagaagt	750	Cloning
pSPL3COL11A1-c.2461-2A>G-R	tcagtcagGGATCCgatgatggg tttgagtggaatgt		
pSPL3COL11A1- c.3204+5G>C-F	ctgactgaCTCGAGtataagaccgttagctccgttga	820	Cloning
pSPL3COL11A1- c.3204+5G>C-R	tcagtcagGGATCCttccaatcatccaaacccttctg		
pSPL3COL11A2- c.4134+1G>A-F	ctgactgaCTCGAGctccatgtcctcttggcccttctc	600	Cloning
pSPL3COL11A2- c.4134+1G>A-R	tcagtcagGGATCCtactacaggaggggcagtct		
COL11A1-ex35-F	tcaacagaagagcaaagtaaaatagc	250	PCR and sequencing
COL11A1-ex35-R	gatgaggtgctagagatggaaattt		
COL11A1_ex24_F	agggtcatcctgggaaagaag	266	PCR and sequencing
COL11A1_ex24_R	aatatgcacgtattcctagccatgg		
